# Editorial: Challenges and outcomes of complex endovascular aortic repair

**DOI:** 10.3389/fcvm.2024.1379282

**Published:** 2024-03-26

**Authors:** George Kouvelos, Konstantinos Spanos, Wolf-Hans Eilenberg, Tilo Kölbel

**Affiliations:** ^1^ Department of Vascular Surgery, Faculty of Medicine, School of Health Sciences, University of Thessaly, Larissa, Greece; ^2^ Department of Surgery, Division of Vascular Surgery, Medical University of Vienna, Vienna, Austria; ^3^ Department of Vascular Medicine, German Aortic Center, University Heart and Vascular Center, Hamburg, Germany

**Keywords:** aneurysm, aorta, dissection, TEVAR, EVAR, complex aortic aneurysm

**Editorial on the Research Topic** Challenges and outcomes of complex endovascular aortic repair

Aortic disease constitutes an important spectrum of arterial diseases including aortic aneurysms (aortic arch, thoracoabdominal and abdominal aorta), acute aortic syndromes (aortic dissection, intramural hematoma, penetrating ulcer and aortic injury), as well as genetic diseases and congenital pathologies (Marfan syndrome, coarctation of the aorta etc.). Endovascular aortic repair has gained wide acceptance in treating patients with complex aortic pathology and has been established as the treatment of choice for most patients with suitable anatomy.

Type A aortic dissection is an acute condition that should be treated by emergent operation immediately after the diagnosis (1). Open repair has been the treatment of choice for most patients. In recent years, hybrid methods have been introduced as a simpler and safer alternative approach. Liu et al. examined the use of ministernotomy in comparison to total arch replacement with frozen elephant trunk in patients with type A aortic dissection. Liu et al. They found that total arch replacement via ministernotomy was safe, feasible, with similar short-term prognosis and also reduced ICU and hospital stay. Patients with type A dissection have a high complication risk. Without intervention, the risk of death raises 1%–2% per hour, while half of patients die within the first 24 h after diagnosis (1). Several biomarkers have been related to higher in-hospital mortality for patients with aortic dissection, showing an incremental prognostic value that can aid in decision making (2). Xie et al. explored the predictive value of preoperative nutritional index (PNI) combined with D-dimer in patients with acute type A dissection. Both markers were independent predictive factors for adverse events during hospitalization. Additionally, Miao et al. found an increase in a certain secreted frizzled-related protein in patients with acute aortic dissection that also may affect the prognosis of the disease.

Long term durability of thoracic endovascular repair depends on the preservation of an adequate proximal sealing zone. The 2020 SVS guidelines support that preoperative or concurrent left subclavian artery (LSA) revascularization is necessary for elective TEVAR (3). Ye et al. reported 83 patients undergoing fenestration or chimney technique to preserve LSA during zone 2 TEVAR for dissection. Complete thrombosis in the aorta distal to the stent graft was above 60% in both techniques with high technical success Ye et al. One of the worst complications after TEVAR is the occurrence of retrograde type A aortic dissection (RTAD) (4). RTAD may occur rarely but may be associated with a high mortality rate (5). Wang et al. reported on 1688 TEVAR patients and found that RTAD occurred in 1.1% of the patients during the procedure, at early and at late postoperative periods. Stent graft oversizing ratio was the only relevant risk factor for RTAD Wang et al. Additionally, aberrant right subclavian artery (ARSA) constitute the most common congenital anomaly of the aortic arch, arising from the proximal portion of the descending thoracic aorta and affecting 0.5%–1% of the population (6). The presence of an aberrant right subclavian artery in patients with type B aortic dissection undergoing TEVAR may affect outcome as the primary tear is often located near the aortic isthmus and the ARSA. Zeng et al. reported their experience in 9 cases and found excellent technical success rate and minimum complications with a mean follow-up period of three years.

The presence of intramural hematoma (IMH) in the proximal sealing zone has also been associated with a worse outcome. Lescan et al. examined two groups of 84 patients with and without intramural hematoma at the proximal landing zone undergoing TEVAR for acute and subacute type B dissections. Migration >10 mm and bird-beaks (failure to achieve a complete proximal seal on the inner curvature of the aorta presenting as a wedge-shaped gap between the stent graft and the aortic wall) (7) were comparable in both groups, while the presence of the IMH may not be relevant to the occurrence of the retrograde type A dissection as a complication after TEVAR Lescan et al.

In complex aortic repair, fenestrated and branched endovascular aortic repair (F/BEVAR) have been efficient and safe in treating patients with supra/pararenal aneurysms (8, 9). However, further advances are needed to decrease the associated morbidity and mortality that accompanies these complex procedures. Kapalla et al. showed that inner-branch technology may provide a feasible therapeutic option in these patients, depicting a high technical success (100%), excellent estimated survival (95%) and branch patency (98%) during a 1-year mean follow-up. Additionally target vessel patency after the procedure is an important factor contributing to the technical and clinical success of F/BEVAR. Loss of patency especially for the superior mesenteric artery (SMA) may become fatal. Gallitto et al. reported their experience with the fate of SMA incorporated during complex aortic procedures. Relining of the SMA stent graft was necessary in 21% of the cases, while SMA orientation determines the necessity of such approach. An aortic diameter >35 mm at the SMA level predicted SMA related adverse events.

Type II endoleak (ET II) has been defined as collateral retrograde flow from the aortic branches. Although half of ET II will spontaneously resolve during follow-up, the sac will increase in up to 25% of patients (10). ET II is considered a benign condition in most cases, however its persistence has been associated with sac expansion and adverse events during the follow-up period (10). Chen et al. examined the association between ETII and the iliolumbar artery and identified the number of lumbar arteries and inferior mesenteric artery diameter as independent risk factors for ET II. Reinterventions for the persistent ET II did not affect long term survival or increased aneurysm related mortality after EVAR.

Coarctation of the aorta is a congenital pathology that has been associated with severe cardiac complication if left untreated. Patients with coarctation of the aorta should be followed on a regular basis despite surgical repair. Zhao et al. identified several risk factors for recurrence after surgical repair including preoperative z-score of the ascending aorta and transverse aortic arch, an arm-leg systolic pressure gradient. Patients with these factors may need close surveillance especially within the first postoperative year.

Advanced endovascular techniques and specialized devices are making endovascular repair a viable option in treating complex aortic pathology. As those techniques and experience with the endografts progress, and the durability of the repair is established and improved upon, we expect that endovascular means will be used more frequently as the treatment of choice for patients with any complex aortic disease.
